# Legal prerequisites for ecosystem-based management in the Baltic Sea area: The example of eutrophication

**DOI:** 10.1007/s13280-015-0656-6

**Published:** 2015-05-28

**Authors:** Annika K. Nilsson, Brita Bohman

**Affiliations:** Faculty of Law, Uppsala University, Box 512, 751 20 Uppsala, Sweden; Faculty of Law, Stockholm University, Universitetsvägen 10C, 106 91 Stockholm, Sweden

**Keywords:** Law, Baltic Sea, Ecosystem approach, Adaptive governance, Social–ecological resilience, Comparative law

## Abstract

The purpose of this paper is to discuss the role of law in the management of the Baltic Sea, with focus on eutrophication. It aims to identify legal instruments or structures realizing an ecosystem approach. This also includes a discussion of the prerequisites of law as contributor to ecosystem-based management (EBM), as well as evaluation of current legal instruments. While ecosystem approach to environmental management is central to contemporary environmental management policy, it is still unclear what such an approach entails in concrete legal terms. The scope of the analysis stretches from international and EU legal regimes, to implementation and regulation within the national legal systems. A conclusion is that the management structures need further development to properly realize EBM, for example, through concretization of management measures, and clarification of duties and responsibilities for their realization.

## Introduction

Over the past decades, the environmental situation of the Baltic Sea has degraded, especially regarding eutrophication, primarily caused by pollution from industry, wastewater, forestry, and not least agricultural practices (HELCOM 2013a). In order to accomplish substantial reduction of pollution in the Baltic Sea area, the coastal states have adopted the Helsinki Convention for the Protection of the Baltic Sea Environment (the Helsinki Convention) and established its administrative organization, the Helsinki Commission (HELCOM). All the Baltic Sea coastal states except the Russian Federation are member states of the European Union (EU), which means that management of the Baltic Sea environment is regulated also by EU law. The main EU legal instruments are the EU Marine Strategy Framework Directive 2008/56/EC (MSFD) and the EU Water Framework Directive 2000/60/EC (WFD). The EU is also a party to the Helsinki Convention. The different legal regimes require the Baltic Sea coastal states to implement and enforce legal measures on a national level to abate eutrophication, mainly through rules aiming to minimize the release of discharges to coastal areas and eventually to marine waters. They also aim to take an ecosystem approach to environmental problems. This paper departs from the question of how the ecosystem approach is reflected in these international and regional legal regimes, and whether such approach is realized in regulatory management of marine environment on the national level. While an ecosystem approach to environmental management is central to contemporary environmental management policy and ecosystem-based management (EBM), it is still unclear what such an approach entails in concrete legal terms.

The paper introduces a discussion of the role and function of law in the EBM of the Baltic Sea. The aim is to investigate what EBM entails in a legal context and how an ecosystem approach could be realized in law. Regulatory management aimed at the problem of eutrophication has been chosen as study example.

In the following section on ecosystem approach, some of the features that characterize EBM are identified and discussed in a regulatory perspective, in order to investigate what EBM entails in a legal context. The basis for this analysis is found in the regulation and further development of the concept of ecosystem approach in official documents. The analysis is further developed by linking to theories on resilience in social–ecological systems (SES resilience). The study does not aspire for exhaustive definition of ecosystem approach or EBM but identification and analysis of some central features in regulatory context.

In the sections on EBM in international and national policy and law, the relevant international, regional, and national regulatory instruments are investigated to find out if and to what extent they reflect regulatory features of EBM. This investigation moreover shows what legal implementation of an ecosystem approach might entail, and introduces a discussion of the potential and prerequisites of law to contribute to EBM and SES resilience. This discussion is based on investigation of regulatory features in the analysis of international, regional, and national law. The analysis includes identification of legal mechanisms or structures that, through their role in EBM, can be presumed to realize an ecosystem approach. The discussion also serves as a basis for a critical analysis of their fundamental prerequisites. Environmental management involves a multitude of different management strategies and instruments, but this investigation aims to identify meaning and formulation of EBM in the legal context, and thus focuses on regulatory management. The role and function of law, in comparison to other manners of governance, is to establish necessary institutional structure and to provide normative steering and authoritative control. The instruments and structures must display clarity and foreseeability, and clear prescription of regulatory powers, including sanctions and compliance control, in order to function appropriately in a legal context.

The investigation comprises critical legal analysis of established sources of law and institutional structures of relevant legal regimes, starting with analysis of provisions, design, structures and internal coordination of the international and regional legal regimes, and followed by a comparative legal analysis of relevant regulatory regimes and their implementation and application of ecosystems approach in four states of the Baltic Sea region: Denmark, Estonia, Poland, and Sweden. The comparative analysis is based on country study reports (available at: bit.ly/country_regulations) produced in 2012–13 in the respective countries. The studies focused on regulation of sewage water treatment and agriculture, as central sources of water pollution causing marine eutrophication. In order to investigate and assess the ecosystem approach, the researchers have analyzed the relevant regulations based on common questions, namely if the studied regulation is based on and aimed at relevant ecological status, adaptive to qualitative spatial and temporal differences or changes, and flexible so as to ensure response to poor ecological status? These questions link to features of regulatory EBM, such as adaptability and flexibility in relation to ecosystem status, multilevel governance, and compliance control, which will be developed next.

## Ecosystem approach

The legal instruments for environmental protection in the Baltic Sea are committed to apply an ecosystem approach, i.e., to provide a basic structure for EBM. This section describes the concept and discusses how it can help identify regulatory features that characterize or support EBM. The ecosystem approach has developed as a concept within international law and was adopted through the Convention of Biological Diversity (CBD) in 1995 (CBD II/8). The interpretation and application of the concept has since then been elaborated. The CBD has developed principles and guidelines to provide a basis for understanding the concept (CBD V/6 2000; CBD VII/11 2004). These elaborations also provide the basis of the concept as adopted and understood within both the EU and HELCOM. HELCOM specifically mentions the concept as it is stated within the CBD in the background documents of its own implementation (HELCOM 2003, 2006).

Ultimately, according to the CBD, the ecosystem approach “…is a strategy for the integrated management of land, water and living resources that promotes conservation and sustainable use in an equitable way…” (CBD V/6). Moreover, the ecosystem approach “…requires adaptive management to deal with the complex and dynamic nature of ecosystems and the absence of complete knowledge or understanding of their functioning. Ecosystem processes are often non-linear, and the outcome of such processes often shows time-lags.” (CBD V/6). Adaptive management is important for the interpretation of the ecosystem approach concept, as it is also the basis of EBM. Furthermore, here it reflects an instrument for the precautionary principle (PP) as part of management of ecosystems also in the absence of complete knowledge. This means that we need to create sustainable structures for management despite lack of full knowledge, and that the design of these structures shall take ecosystem dynamics as a fundament and thus need to be adaptive. It is also explicitly stated that “…approaches, which are also relevant to other environmental conventions, including ‘ecosystem based management,’ [and] ‘integrated river-basin management’…may be consistent with the application of the Convention’s ecosystem approach, and support its implementation in various sectors or biomes” (CBD VII/11). It is interesting to note the direct relationship between EBM and ecosystem approach. Integrated river basin management is adopted in EU law on water management, primarily through the WFD, which could be considered a management structure coherent with the ecosystem approach. It is also noteworthy that “…an ecosystem approach…recognizes that humans, with their cultural diversity, are an integral component of many ecosystems.” (CBD V/6). In the latter citation, the integral part that humans play is held as a principal point, which is also reaffirmed in the BSAP preamble (HELCOM 2007). The statements cited above reflect a perspective on nature and ecosystem management, which could be interpreted as reflecting the view of ecosystem management discussed within research on SES resilience. SES resilience can therefore contribute to the understanding of the perspectives presented within the CBD on the ecosystem approach.

Management principles elaborated by the CBD contain a number of statements relevant for the application of the concept. They also clarify the objectives and aim of ecosystem approach, and present a number of basic points of departure for management. One basic point is that ecosystem approach must be pursued through adaptive management in order to anticipate and cater for ecosystem changes and events. The principles moreover state that the ecosystem approach should be undertaken at the appropriate spatial and temporal scale, which is also emphasized within resilience research. Management should be decentralized to the lowest appropriate level and involve all stakeholders. The varying scales and lag-effects that characterize ecosystem processes imply that objectives for ecosystem management should be set for the long term (CBD V/6).

References to SES resilience theories are detected in the elaborations of the concept of ecosystem approach under the CBD, as described above (CBD V/6). Research on SES resilience provides a framework for EBM and the intertwined connection between social and ecological systems in this regard, i.e., the integrated role of humans. It reflects an initiated discussion on how and why certain features of EBM are important, and thus helps identify relevant regulatory features that match. SES resilience theory is therefore used to provide more concrete reasoning in regard to relevant management features of an ecosystem approach. The SES resilience theory and management features relevant in this perspective are reflected in a collective picture from a number of sources (see e.g., Folke et al. 2002, 2005; Berkes et al. 2003; Dietz et al. 2003; Folke 2006; Walker and Salt 2006).

Some of the features reflected in SES resilience theory are flexibility, adaptability, and operating on the relevant temporal and spatial scales. These features are to be understood as mechanisms that primarily respond to the dynamics and inherent characteristics of ecosystem functions. Adaptability in this regard signifies features that embrace and impose new knowledge into the management activities: features with the ability to follow and adapt to both the changes within the ecosystem, as well as to the technological, scientific, or other changes in the social system. Flexibility signifies management activities that correspond to spontaneous and fast changes in the ecosystem, and includes mechanisms that are open to change and not linear-bound, and management actions that are flexible and adjustable to different situations. These features are connected to the different temporal and spatial scales. The management activities will depend on the spatial scale chosen. Generally, a larger scale needs less of flexibility, as flexibility is a faster response mechanism than adaptability, and the other way around. Most environmental problems need to be addressed by management activities at many different scales in parallel. Baltic Sea eutrophication is an illustrating example, as there are a multitude of causes and actors involved, which necessitates management at regional, international, and national scales. The need for parallel actions is also represented by features of EBM, according to resilience research, such as having a multilevel approach to management, a wide participation in management tasks and decision-making, to create and pursue a diversity of measures, and to have clear paths to enforcing compliance.

In recent years, the connections between SES resilience and law have been discussed within legal research (see, e.g., Ebbesson and Hey 2013; Garmestani and Allen 2014). In many ways, the described resilience features resemble mechanisms or structures that can be found within law—with a somewhat different role or function due to the specific strictness necessary within a legal system. The legal system has an important role in steering human activities and hence affects the interactions in social–ecological systems. In order to be effective in this task, the legal system must also incorporate certain features within its structures, features that correspond to the complex dynamics of the ecosystem, i.e., features of ecosystem approach and EBM discussed above. It is necessary to further investigate to what extent law is compatible with such features and what specific implications appear within the legal sphere.

## Ecosystem-based management of the Baltic through international and EU law

Many features of EBM are represented within the environmental legal regimes in the Baltic Sea area. This section will investigate the prerequisites in international and EU levels of regulation in the Baltic Sea area, for realizing EBM. These levels of regulation form the foundation on which coastal states base their national laws, and thus, the extent to which they show potential to promote effective EBM is important.

As a starting point, international environmental law structures inherently provide space for both flexibility and adaptability. The flexibility is primarily a result of the considerable room for discretion left to the states in determining how to implement an agreement. Flexibility also comes with the formulation of requirements in international agreements, which are often vague and thus provide a wide range of possible interpretations and ways of implementation. The main obligation of the Helsinki Convention, for example, states that the parties shall “…take all appropriate legislative, administrative or other relevant measures to prevent and eliminate pollution in order to promote the ecological restoration of the Baltic Sea Area and the preservation of its ecological balance (article 3(1)).” This is followed by paragraphs stating mainly that in their efforts to eliminate pollution, the general principles of environmental law should be applied. Although the general provisions of international law lack adaptability or flexibility in being hard to change once adopted, adaptability within international agreements is reflected in procedures for adopting annexes or protocols, as a mechanism to review, enhance, or up-date an agreement with more accurate knowledge. Such mechanisms are found in the Helsinki Convention, where general provisions are combined with a possibility to adopt annexes with details on how to interpret the requirements. Moreover, recommendations are continuously issued by HELCOM with guidance on how to apply the Helsinki Convention.

The international law system is increasingly complex and multileveled, due to an increasing amount of international agreements being adopted (see for example Kim 2013). In the Baltic Sea area, this development is seen in the parallel application of the fundamental UN Convention on the Law of the Sea (UNCLOS), the Helsinki Convention, the BSAP, and a large number of EU directives. They form an overlapping net of intertwined multidimensional regulatory frameworks for the protection and preservation of the marine environment of the Baltic Sea. This reflects important features of EBM providing a basis for a diversity of measures to abate environmental degradation, and creating instruments or mechanisms adjusted to the appropriate spatial and temporal scales. These features aim to increase the potential to solve the problem. From a legal point of view, however, this can create obstacles for effective implementation and compliance review. The interconnectedness and multitude of regimes, in addition to the flexibility provided, could make effective compliance control difficult. The reasons for this will be elaborated further on. It should, however, be noted as a prerequisite that the legal system has specific characteristics in comparison to management in general. The enforcement of measures through compliance control is a significant aspect of law. It is significant since this role of law reflects the potential to push or pressure states into a wanted behavior, as a result of implementation of required measures, and increases the potential to reach the environmental goal. This is a function of law that separates it from management in general, and that can also contribute beyond other management structures. The complex dynamics and diversity that provide prerequisites for EBM could thus create an obstacle to effective management. Still, regardless of this potential conflict, if successful coordination between these overlapping and parallel regulations can be accomplished, then there is potential to create a strict, adaptive, and effective regulatory platform.

One way of incorporating flexible and adaptive features in an international environmental agreement is through general principles of environmental law. These principles, such as the precautionary principle (PP), the polluter pays principle (PPP), the principle of best available technique (BAT), and the principle on best environmental practices (BEP), have developed in response to the need for proactivity and precaution in relation to the environment. They are all included in the provisions of the Helsinki Convention. The fact that interpretation of the environmental law principles changes with the continuous changes in the environment, as well as with technological and scientific developments, makes them adaptive. Their flexibility lies in the general applicability that does not narrow down any precise way to implement them.

In recent years, the EU has gained increased importance as an actor in the governance of the Baltic Sea. The WFD, adopted in 2000, was one of the first more holistic directives focusing on water governance, but then only within the member states applying mainly to internal waters, but including “…surface water on the landward side of a line, every point of which is at a distance of one nautical mile on the seaward side from the nearest point of the baseline…” (WFD Article 2(7)). It includes a number of more area specific directives to be involved when the member states establish river basin management plans with measures for good ecological status (GES) by 2015 (articles 4 and 13). GES then represents an environmental state based on scientific knowledge and defined by different environmental goals (see definitions in articles 2(17–25)). The MSFD is complementary in scope to the WFD and was adopted in 2008. It has been significant for the joint management efforts in the Baltic Sea area. The MSFD is applicable to marine waters, i.e., the waters in the territorial and exclusive economic zone beyond internal waters (article 3(1)a), including coastal waters on the seaward side of the baseline. The MSFD thus overlaps the scope of the WFD by one nautical mile. The MSFD aims to achieve GES by 2020 (article 1). One of the quality descriptors determining GES according the MSFD is that human-induced eutrophication shall be minimized (article 9 and Annex I). The states are required to individually adopt the so-called marine strategies (article 5). Aside from such general obligation, the MSFD does not specify the requirements, or measures, it imposes on the states. It is instead goal-oriented, as the strategies should contain measures that the states deem necessary to achieve GES. It thus leaves exceptionally large space for states to decide on measures for implementation, in comparison to directives in general. A significant feature of the MSFD is that it calls on the member states to use “…existing regional institutional cooperation structures, including those under Regional Sea Conventions, covering that marine region or subregion” (article 6) in order to achieve the coordination intended. This was one of the reasons to why HELCOM adopted the BSAP in 2007. The BSAP aims to reflect the MSFD but with specific focus on the Baltic Sea region. The BSAP also aims to achieve GES and defines such environmental objectives. Thus, the WFD, the MSFD, the BSAP, and indirectly the Helsinki Convention are instruments that are parallel, intertwined, and inter-connected in a unique way, and that has consequences for their interpretation and implementation. Goal-oriented regulatory regimes focusing on environmental status, as the legal instruments regulating Baltic Sea environment, provide examples of adaptive and flexible legal structures signifying features of ecosystem-based regulation. As described, this form of regulation provides ample space for states to adjust their implementation to national circumstances, but with a clear and qualitative environmental goal. Using environmental status as a basis is a fundamental step in applying an ecosystem approach. In combination with the features of flexibility and adaptability, it creates important prerequisites for imposing EBM.

In a procedure similar to the marine strategies of the MSFD, the states are in accordance with the BSAP to decide on national measures independently and report them to HELCOM in National Implementation Plans (NIPs) (HELCOM 2007). In contrast to the MSFD, the BSAP contains reduction targets for each state, which represent the level of discharge reductions needed to achieve GES in the Baltic Sea (HELCOM 2007). The reduction targets are not binding but nonetheless important, as they express necessary discharge reductions, and hence the extent of action and measures needed. From a legal perspective, the focus on environmental status is an important complement to the more vague statements found in the Helsinki Convention. It is important as it provides orientation to the definition of appropriate and relevant measures, and does that even more in the light of the reduction targets. The reduction targets also raise the bar in relation to the guidance provided in recommendations and annexes, and can provide incentives to issue more updated versions corresponding better to the level of discharge reductions necessary. As the BSAP aims to reflect the MSFD and the regional definition of GES, the reduction targets should be taken into account also in the implementation of measures in accordance with the MSFD. In this aspect, the diversity of regulations and possible measures create an interesting dynamic regulatory situation, where the different instruments are strengthened by each other and mutually provide substance. The flexibility in adopting measures and the structure for how to do that also provide potential to create the kind of diversity that characterizes EBM.

In contrast, it is stated that for EU member states, the WFD river basin plans can be incorporated in the NIPs (HELCOM 2007), although the scope of the WFD is not the same as that of the BSAP or the MSFD. It could be argued that the activities that need to be regulated are to a large extent the same, but still, the goal of the WFD only concerns internal waters. This implies that it would be inadequate to only implement the WFD river basin plan to meet the goals of the BSAP. Presumably, this statement within the BSAP aims for increased coordination as well as to lessen the burdens on the states. Despite the difference in scope, the states seem to interpret the statement within the BSAP as if compliance with the BSAP only requires implementation of the WFD river basin plans. It seems as if states mainly implement measures that they are already bound by. As a result, few new initiatives for measures and management actions have arisen (HELCOM 2013b), despite the fact that the reduction targets of the BSAP, in connection to the MSFD, necessitate further action and additional measures. It might be that the flexibility provided gives the states leeway for less strict implementation.

As has been implied, the interconnectedness and multitude of regimes, in addition to their flexibility, make the possibilities for effective compliance control difficult. What is compliance or not becomes unclear since the legal instruments only to limited extent specify what the states are required to do. Effectiveness of a legal instrument can of course be assessed in different ways. It is not necessarily directly referable to strict compliance control. Effectiveness must ultimately be assessed in relation to whether the goal aimed for with the regulation imposed is accomplished (see Bodansky 2010). However, in a legal system, compliance control provides means to assess such development. Contrary to HELCOM, the EU, as a supranational regime, has significant tools and competences to act strictly on non-compliance. Cooperation between the EU and HELCOM could therefore be important. However, as the nature of the environmental problem in itself is diffuse and difficult to estimate in terms of results, this adds additional insecurity regarding control measures and the ability to define whether the measures taken by the states are adequate. Circumstances with uncertain requirements might create inefficiency in the legal governance of the Baltic Sea as it then lacks ability to force states towards change. Somehow compliance control, or ensuring efficiency, must contribute to coherence in implementation, which will possibly also promote willingness to comply, create incentives for further action, and increase the potential for positive results.

## Ecosystem-based management of Baltic Sea environment in national law and policy

### National policy and law instruments to abate marine eutrophication

International law, including EU law, on Baltic Sea environment thus provides legal basis and framework for regulatory EBM. Implementation and realization of such management must, however, take place on national and local level. In this section, features of EBM in Danish, Estonian, Polish, and Swedish law are analyzed in comparison, and the prerequisites of national law in such management are discussed. The comparison is based on country studies conducted in 2012–13, and thus relates to valid law in 2012–13.

Marine environment is central to Swedish environmental policy, and eutrophication is held as an acute and prioritized problem (Nilsson 2013). Danish and Polish environmental policies also state commitment to marine environment (Baaner and Tegner Anker 2013; Nyka 2013). Marine eutrophication is, however, rather invisible in Estonian policy, except for the BSAP implementation plan, prescribing quite detailed measures regarding wastewater treatment and agriculture. The Estonian Environmental Strategy 2030 states measures taking water status more into consideration, but deals with marine issues only in very general terms (Broks et al. 2013).

Fulfillment of international and EU law obligations, as well as implementation of national policies on Baltic Sea environment and water management, is to great extent achieved through regulatory measures. An ecosystem approach calls for multileveled and multistakeholder procedures and diversity of methods, but regulation is still a prominent management method. Regulation of sewage water and agriculture with regard to nutrients pollution are linked to requirements in the Helsinki Convention and the EU Urban Waste Water Directive 91/271/EEC, and show similarities in the studied legal systems. Large sewage treatment plants are subject to permit regulation, meaning that they need concession from a public authority, allowing and conditioning operation of the installation, including individual technical requirements and emission limits for the specific installation in accordance with best available technique (BAT) and in relation to its affected environment. Permit regulations are generally open to review or even revocation when motivated by environmental changes, technical development, or new knowledge. A permit procedure is thus a process with potential for adaptability through individual regulation based on scientific evidence and ecological standards. Substantive environmental demands are weighed against other—mainly economic—interests, which may lead to permit conditions that are insufficient from environmental perspective. A pragmatic approach is reported in some studies. In Denmark, a 1998 report showed that a large number of plants did not comply with their permits. More recently, the Nature Agency reported that the stricter emission standards are required of treatment plants, the more are permit conditions violated. They therefore recommended a more appropriate regulatory climate, in which permit regulation is set at a level that is more realistically achievable in the long term. (Baaner and Tegner Anker 2013).

The studied legal systems show similarities also in regulation of nutrients emissions from agriculture. There are comprehensive best environmental practice (BEP) norms on, for example, storage of manure, limiting amounts on nutrients applied through fertilizers, as well as periods, conditions, and methods for their application in order to prevent nutrient losses. These norms implement the Helsinki Convention and the EU Nitrates Directive 91/676/EEC, etc. BEP norms are prescribed not only in regulation, but also in recommendations that are formally not binding. BEP are also promoted through economic policy instruments (see Baaner and Tegner Anker 2013; Nilsson 2013). Some agricultural activities are subject to permit regulation as polluting activities—but here national regulations vary. Very large livestock farms are regulated through industrial emissions permits under EU law through the Industrial Emissions Directive 2010/75/EU (IED), thus also requiring environmental impact assessment. In Denmark, and to some extent Sweden, we see a wider scope of the regime, sometimes with simplified permit or notification procedures for the smallest farms.

Somewhat different experiences of legal compliance have been reported. In Estonia, levels of compliance are uncertain, due to inadequate systems for monitoring and control. Many breaches identified via monitoring are not further processed because supervisory authorities do not have swift access to further monitoring data. Moreover, sanctions for misdemeanors concerning agricultural activities are not sufficiently strict (Broks et al. 2013). In Poland, reoccurring shortcomings in compliance are noted (Nyka 2013). In Denmark, public statements report generally good legal compliance (Baaner and Tegner Anker 2013). It is, nevertheless, clear that the national systems have not seen sufficient results with regard to mitigated eutrophication problems. Political challenges of overregulation are reported, especially in relation to agriculture (see Baaner and Tegner Anker 2013; Nyka 2013).

Water management systems based on the WFD have been introduced in all the studied legal systems, thus establishing comprehensive institutional structures for monitoring, assessment, and planning for relevant and ecosystem-based water management. A fundamental function of the system is to provide coordinated and comprehensive ecosystems approach to different management measures in different sectors of policy and regulation.

### Implementation and realization of EBM in the national legal systems

Even though declarations of ecosystem approach can be found in national policy documents of the Baltic Sea region, there is no clear implementation of the concept. It is reported, for example, that the ecosystem approach is not recognized in the Polish legal system, and that there is a long way to go before it will be (Nyka 2013). However, in all the compared countries, aspects of ecosystem approach have been introduced at a more strategic regulatory level. The following part provides analysis of the potential and application of such approach, based on earlier provided indicators.

An ecosystem approach has been introduced in the studied legal systems through implementation of the WFD. The water management systems ideally provide a comprehensive and combined approach to water management and require more stringent regulation when necessary for achieving environmental objectives set out in the relevant legislation and management plans. The strategic policies and plans are, however, reported as not yet sufficiently integrated, so as to provide comprehensive and combined management linked to concrete and differentiated management measures (see, e.g., Baaner and Tegner Anker 2013; Broks et al. 2013). It is reported that the water management policies and plans are not clearly integrated with marine environment policy. National implementation of the MSFD seems in early days. Often there is no comprehensive regulation on management, protection, and use of the sea established in national law (Baaner and Tegner Anker 2013; Broks et al. 2013). Poland’s implementation of the MSFD is reported as a failure, introduced only in 2013, after the EU Commission brought infringement action against Poland (Nyka 2013). After Poland’s further implementation steps, the infringement procedure was closed, 23 July 2013. In Sweden, EU marine policy is a point of departure for the national marine policy, and coordination of the MSFD, BSAP, and WFD systems is claimed necessary for effective management. A marine planning and management system has been established to provide infrastructure for comprehensive EBM of marine resources, but has had little practical use in regulatory management action so far (Nilsson 2013). In Denmark, the Act on Marine Strategy lays duties on public authorities to take into account its objectives and action programs when exercising administrative powers through, for example, issuing permits or enforcement orders. The act does not provide competences to intervene in on-going lawful activities, nor does it impose obligations to act as prescribed in marine strategy action programs. It is reported that reduction in land-based emissions of nutrients into the sea is not managed here, but expected to follow implementation of WFD management plans, with positive effects on marine waters (Baaner and Tegner Anker 2013). These comparative results show that water and marine management systems with aims and potential for pluralistic, intertwined, and multileveled governance have been introduced on a national level, but that they have not yet been fully realized. There is also some indication that the interconnectedness of the different regimes could hinder appropriate implementation and integration of marine ecosystem-based management, similarly as observed earlier in the international context.

Similarly to what has been observed in regard to international and EU law, the extensive flexibility of legal regimes makes effective compliance control difficult also on a national level. The comparative study has shown uncertainties regarding the legal status of management plans and action programs. A fundamental legal prerequisite is that the law specifies legal duties and responsibilities, i.e., what precisely must be done—or achieved—who must do it, who controls that it is done, and what happens if it is not. It is unclear if the national management plans and action programs must be followed, and if and how inadequate implementation and compliance can be controlled and sanctioned. In Estonia, implementation of the WFD (and other EU law, including the MSFD) is ensured through minimalistic and verbatim transposition, mainly in the Water Act, of the generally formulated text of the directive. This entails implementation of statement and indicators of an EBM approach, but not necessarily an operational ecosystem approach, as necessary prerequisites for ensuring effective implementation are missing. For example, the Water Act does not specify how flexible management measures should be taken, or who should take them, which in practice makes the national legal framework useless (Broks et al. 2013). The normative status of quality objectives is also often unclear. Water status objectives must be made operational in order to function in the regulatory system. In Sweden, quality objectives can be specified in legal quality norms, but such specified norms have not been established for ecological status. Even if they had, these norms are formulated and perceived as ‘objectives’ rather than ‘limit values,’ which means that they are applied as less absolute and not directly enforceable in individual cases (Nilsson 2013). Again, the normative status of these legal documents and rules is low—or flexible, which makes legal compliance control challenging. Moreover, much room is left to regulatory discretion, and the limits of such discretion are even more difficult to control.

In Sweden, there is a comprehensive institutional structure for ecosystem-based adaptive water management. Management measures are carried out through existing regulation in different sectors, for example, permit regulation of treatment plants or administrative supervision and regulation of farms. Adaptive ecosystem management is to be realized with support of the water management system, directing regulators in a more coordinated manner to reach objectives of adequate environmental status, including stricter regulation of individual actors when necessary, but it is unclear how this is to be implemented and controlled. Such management approach is much left to the administrative discretion of the regulator (Nilsson 2013). It could therefore again be argued that the institutional design crucial for the functioning of the regulatory management system is not sufficiently clear or strong.

In comparison, the Danish water management framework was reported to establish a more specified and authoritative system at the time of the country study. River basin management plans are to be drafted by the Nature Agency and enacted by the Minister of Environment, as authoritative and comprehensive regulatory instruments, designed with a legally binding section and an explanatory section. The legally binding section comprises environmental objectives for individual bodies of water, action programs, and a set of administrative guidelines that function as instructions directed to the administrative authorities. Municipalities shall further develop municipal water action plans for how the plans’ action programs shall be implemented locally. The plans and programs are reported to interact with existing regulations and build on existing measures, and to function as significant part of implementation of the WFD, as well as for meeting the environmental objectives of the Nitrates Directive concerning agricultural nitrates pollution, and to some extent the MSFD. The measures are implemented by amending existing and developing new legislation, and through exercise of administrative powers of the competent authorities—as authoritatively directed in the administrative guidelines of the plans. This order is similar to the Swedish system, where the plan only identifies the necessary measures and directs other authorities to take them. The Danish plans are, however, formally and clearly legally binding. The instructions and administrative direction has considerably more authority, as they are decided by the minister and directed to his or her subordinate administrative bodies (Baaner and Tegner Anker 2013).

A general observation is that there is room for adaptability in national regulation in the differentiation of general rules or individual regulation. General rules will, for example, set more stringent requirements on wastewater discharges in sensitive areas, in accordance with EU law. Individual regulation, such as permit regulation, shows potential for adaptive management through individual regulation that should be site specific, based on gained knowledge of the relevant environment. Such regulation should be based on flexible general environmental principles and goal-oriented regulation focused on environmental status. In practice, however, it is not possible to identify any greater extent of adaptability in wastewater management regulation. It seems there are only weak and sparse direct links between the ecological status of the Baltic Sea ecosystems and regulation of wastewater treatment (see, e.g., Baaner and Tegner Anker 2013). In the Estonian Water Act, it is possible to set up to 30 % stricter limit values for pollutants in wastewater than those stipulated in general regulation, if the status of the recipient water body is not good, or if there is a risk that it may deteriorate. In practice, stricter norms have been set in a few cases, but it is reported that permit authorities will generally not resort to stricter provisions, as causality between relevant emissions and the deteriorated status must be proven with sufficient certainty (Broks et al. 2013).

Regulatory flexibility and responsiveness in the context of a deteriorated or otherwise changed environmental situation seems rare and left to the authorities’ administrative discretion. Necessarily large cuts in emission standards for Swedish municipal sewage treatment plants are argued as unrealistic. There is no political acceptance for sufficient cuts in general regulation, and it is stated that radical amendments in current permit conditions will be deemed unreasonable. Permit reviews are, moreover, rarely initiated, as few competent authorities can devote resources necessary for such review, at least on any regular basis (Nilsson 2013). Estonian law contains grounds and competences for refusing environmental permits or reviewing existing permit conditions in view of breached environmental quality standards. However, such flexibility is rarely seen in practice, as the lack of adequate monitoring and knowledge makes it hard to prove causality between the activity and the environmental problem (Broks et al. 2013). It is also reported that regulatory control of the majority of agricultural activities not regulated by an IED permit is lacking. There are no legal grounds for regulating obligatory farm-specific environmental requirements that go further than the basic general rules. Supervisory bodies may only ask the farmer to take supplementary measures on a voluntary basis. There are little regulatory means for responsiveness to a changed environmental status (Broks et al. 2013). All this means that compliance control can be challenging.

The water management system is intended to provide a fundamental comprehensive and combined management approach to coordinate established environmental regulation. Water management should be based on collected data and information about the status of water environment and of sources and activities causing pressure on water environment, and provide a coordinated strategy for appropriate management action through management plans and action programs. It is, however, reported that plans and programs are very generally formulated, and that even though regulation is held as central to the management system, there is little concrete direction of regulatory measures. Statements of action programs in Estonian management plans are described as abstract and going little beyond stating the obvious or declaring that existing legal requirements must be fulfilled. Examples from sub-district plans show statements that it is not possible to assess the effectiveness of the measures at the level of the water body, as most measures are not presented at that level. Furthermore, it is not stated how the measures were developed, who should comply with the plans, and by what time. There is no clear mechanism for ensuring that measures will be taken (Broks et al. 2013; compare to Poland, Nyka 2013). In the Swedish system, it is noted that the measures stated in the action programs have so far been quite generally formulated as guidance to authorities at different levels of administration to take rather self-evident action. The measures mostly focus on improving knowledge and data. For operative administrative authorities, typically local and regional regulators, the action plans state that they should draw up strategies and plans for taking measures in areas not achieving good status, and that they should prioritize such areas in their supervision. The few more specific measures state that local authorities ‘need to’ demand high level of protection for private sewerage that contributes to a water body not achieving good water status, and that regional authorities ‘need to’ make a revision of operations with environmental permits and if necessary move to review such permits. Such prescription of measures cannot be seen as other than very general and non-controllable recommendations to the authorities on considerations to be made when planning their work and when utilizing their administrative discretion (Nilsson 2013).

Another critical observation is that adaptability is not fully realized in management planning. While the management system provides for basin-based measures, the plans often reflect generally applicable state-wide basic measures, for example, in Estonia, where there are no specific numbers of nutrient loads or reduction targets in the respective river basins. The correlation between water status assessment and undertaken management action is reported as insufficient, and the Estonian National Audit Office has proposed that the Ministry of Environment should focus more on ensuring this correlation and to take such observations in account in planning future action (Broks et al. 2013). It can be noted that the action programs for the different Swedish water districts are, for all relevant purposes, identical, stating the same measures for all the different water districts, without differentiation between their respective sub-basins (Nilsson 2013). This similarity cannot be held to manifest adaptive management in the, in some respects, different districts and water bodies, with variation of ecological status and different pressures.

In summary, national water management systems should provide a fundamental and comprehensive ecosystem approach to water management, to support and direct application of adaptive and flexible EBM through established sector regulation of nutrient emissions causing eutrophication of the Baltic Sea. However, while the management systems can provide support to the progressive regulatory authority, they do not consistently provide clear and authoritative guidance and control needed in a regulatory context.

## Conclusions

EBM is crucial for effective environmental management but little has been done to translate what such management implies within legal regulation. The concept of ecosystem approach is an attempt to introduce such management also within the regulatory sphere. It has arisen in international law over the past decades and is today adopted and acknowledged within many international environmental agreements and in EU law. The elaborations of how to apply the ecosystem approach refer to typical features of EBM, such as adaptability and flexibility together with other structural features such as multilevel approaches, taking temporal and spatial scales into account and providing for a diversity of management actions. In this paper, we have explored legal structures and mechanisms that could represent regulatory EBM, and to what extent such features are represented in the legal instruments for the environmental protection of the Baltic Sea.

The Baltic Sea legal regimes generally reflect many features of EBM. International and EU regimes reflect such features both in their inherent structures and in more specific provisions. The Helsinki Convention, for example, includes general environmental principles that are adaptive and flexible. Furthermore, it has mechanisms for up-dating the provisions through annexes and recommendations. More signifying for the Baltic Sea regulatory instruments is complex overlapping ecosystem-focused instruments with goal-oriented approach. Together these structures and mechanisms result in a flexible and adaptive multileveled system, providing for states to implement a diversity of measures, thus reflecting features typically in line with resilience thinking. However, a concern that this raises from a legal perspective is that the complexity and diversity entails difficulties in controlling compliance, which is central in accomplishing the aims of a legal instrument.

Comparative analysis of the national laws has showed that the chosen regulatory techniques were rather similar and that there was space for flexibility and adaptability. Procedures for individual regulation, for example, permit regimes, hold potential for such adaptability and flexibility, in relation to the ecological status and the functioning of the relevant ecosystem. As do also environmental law principles when implemented in planning and legal decision-making nationally. It is, however, noted that the potential was generally not utilized at the time of this study. In order for the procedures to better implement an ecosystem approach, they need to be supported by clear normative structures and control structures. The management structures set up for water management under the WFD aim for ecosystem approach, but at the same time, the legal status of these structures and environmental objectives seem uncertain. Management plans and action programs are also reported as generally formulated without clear direction. This entails uncertainty in their application in regulatory procedures, and in efforts to control and enforce compliance. Moreover, the marine environmental management system still seems poorly integrated in regulatory procedure, and its realization risks being left to existing management systems. In the future, the legal status and meaning of the management plans and action programs—also marine management plans—will also have to be clarified and fortified, and fully integrated in the regulatory system, so that the management authorities’ realization of a coherent and coordinated EBM can be directed and controlled. This should entail, among other things, concretization of the substance and time frame for the management measures, and clarification of duties and responsibilities for their realization.

A general conclusion is that the vagueness that flexible and adaptive features imply challenges the legal systems in controlling compliance at both national and regional levels. However, most of the legal regimes reviewed are relatively new and already creating changes in the regulatory approaches chosen at all levels. As problems are detected and the management systems develop, hopefully it is possible to eventually create a better balance between these different features.

## References

[CR1] Dietz T, Ostrom E, Stern PC (2003). The struggle to govern the commons. Science.

[CR2] Ebbesson J, Hey E (2013). Introduction: Where in law is social–ecological resilience?. Ecology and Society.

[CR3] Folke C (2006). Resilience: The emergence of a perspective for social–ecological systems analyses. Global Environmental Change.

[CR4] Folke C, Carpenter S, Elmqvist T, Gunderson L, Holling CS, Walker B (2002). Resilience and sustainable development: Building adaptive capacity in a world of transformations. Ambio.

[CR5] Folke C, Hahn T, Olsson P, Norberg J (2005). Adaptive governance of social–ecological systems. Annual Review of Environmental and Resources.

[CR6] Kim R (2013). The emergent network structure of the multilateral environmental agreement system. Global Environmental Change.

[CR7] Country reports published digitally on the official web page of Stockholm University and BEAM: http://www.su.se/ostersjocentrum/english/beam/legal-aspects-of-the-ecosystem-approach/country-studies.

[CR8] Baaner, L., and H. Tegner Anker. 2013. Danish Law on Controlling Emissions of Nutrients in the Baltic Sea Region.

[CR9] Broks, K., Relve, K., and Veinla H. 2013. Estonian Law on Controlling Emissions of Nutrients in the Baltic Sea Region.

[CR10] Nilsson, A.K. 2013. Regulating Zero Eutrophication. Swedish Law on Controlling Emissions of Nutrients to the Baltic Sea.

[CR11] Nyka, M. 2013. Polish Law on Controlling Emissions of Nutrients in the Baltic Sea Region.

[CR12] CBD II/8. 1995. The Second Meeting of the Conference of the Parties (COP) of the Convention on Biological Diversity, held in Jakarta, Indonesia. Decision 8. UNEP/CBD/COP/2/19, p. 12.

[CR13] CBD V/6. 2000. The Fifth Ordinary Meeting of the Conference of the Parties to the Convention on Biological Diversity, held in Nairobi, Kenya. Decision 6. UNEP/CBD/COP/5/23, p. 103.

[CR14] CBD VII/11. 2004. Seventh Meeting of the Conference of the Parties to the Convention on Biological Diversity, held in Kuala Lumpur, Malaysia. Decision 11. UNEP/CBD/COP/7/21, p. 186.

[CR15] HELCOM. 2003. First Joint Ministerial Meeting of the Helsinki and the OSPAR Commissions, held in Bremen, Germany, June 2003. Agenda item 6, Annex 5; Statement on the Ecosystem Approach to the Management of Human Activities; “Towards an Ecosystem Approach to the management of human activities”. JMM 2003/4-Rev.1-E.

[CR16] HELCOM. 2006. Ecological Objectives for an Ecosystem Approach. HELCOM Stakeholder Conference on the Baltic Sea Action Plan, Helsinki, Finland.

[CR17] HELCOM. 2007. Baltic Sea Action Plan, adopted at the HELCOM Ministerial Meeting held in Krakow, Poland.

[CR18] HELCOM. 2013a. Review of the Fifth Baltic Sea Pollution Load Compilation for the 2013 HELCOM Ministerial Meeting. Baltic Sea Environment Proceedings No. 141.

[CR19] HELCOM. 2013b. Overview of implementation of the HELCOM Baltic Sea Action Plan. Prepared for the 2013 HELCOM Ministerial Meeting to give information on the progress in implementing the HELCOM Baltic Sea Action Plan.

[CR20] Berkes F, Colding J, Folke C, Berkes F, Colding J, Folke C (2003). Synthesis: Building resilience and adaptive capacity in social–ecological systems. Navigating social–ecological systems—Building resilience for complexity and change.

[CR21] Bodansky D (2010). The art and craft of international environmental law.

[CR22] Garmestani AS, Allen CR (2014). Social–ecological resilience and law.

[CR23] Walker B, Salt D (2006). Resilience thinking—Sustaining ecosystems and people in a changing world.

